# A framework for space-efficient read clustering in metagenomic samples

**DOI:** 10.1186/s12859-017-1466-6

**Published:** 2017-03-14

**Authors:** Jarno Alanko, Fabio Cunial, Djamal Belazzougui, Veli Mäkinen

**Affiliations:** 1 Department of Computer Science, University of Helsinki, Gustaf Hällströmin katu 2b, Helsinki, 00560 Finland; 2 Max Planck Institute for Molecular Cell Biology and Genetics, Pfotenhauerstr. 108, Dresden, 01307 Germany; 3 DTISI, CERIST (Research Centre for Scientific and Technical Information), Rue des 3 Fréres Aissou, Algiers, 16306 Algeria

**Keywords:** Metagenomics, Read clustering, Text indexing, Burrows-Wheeler transform, Suffix-link tree, Right-maximal *k*-mer, Submaximal repeat

## Abstract

**Background:**

A metagenomic sample is a set of DNA fragments, randomly extracted from multiple cells in an environment, belonging to distinct, often unknown species. Unsupervised metagenomic clustering aims at partitioning a metagenomic sample into sets that approximate taxonomic units, without using reference genomes. Since samples are large and steadily growing, space-efficient clustering algorithms are strongly needed.

**Results:**

We design and implement a space-efficient algorithmic framework that solves a number of core primitives in unsupervised metagenomic clustering using just the bidirectional Burrows-Wheeler index and a union-find data structure on the set of reads. When run on a sample of total length *n*, with *m* reads of maximum length *ℓ* each, on an alphabet of total size *σ*, our algorithms take *O*(*n*(*t*+log*σ*)) time and just 2*n*+*o*(*n*)+*O*(max{*ℓ* *σ*log*n*,*K* log*m*}) bits of space in addition to the index and to the union-find data structure, where *K* is a measure of the redundancy of the sample and *t* is the query time of the union-find data structure.

**Conclusions:**

Our experimental results show that our algorithms are practical, they can exploit multiple cores by a parallel traversal of the suffix-link tree, and they are competitive both in space and in time with the state of the art.

## Background

High-throughput sequencing has made it fast and cost-effective to sequence DNA from entire environments at once. The collection of all genomes in an environment is called the *metagenome* of the environment. A fundamental problem in metagenomics is to cluster the reads produced by a high-throughput experiment, according to which species (or, more generally, taxonomic unit) they originate from. This can be done in a supervised manner, by mapping the reads to a database of known genomes, or in an unsupervised way, by performing extensive comparisons of all reads against each other without relying on any reference database. Unsupervised methods are attractive, and in most practical cases the only option available, since the genome of most organisms (e.g. prokaryotes) that inhabit complex environments is unknown.

Having accurate clusters for reads that come from unknown taxonomic units allows one to estimate key measures of environmental biodiversity, and to assemble the corresponding genomes more accurately and using less memory [1–3]. Clusters have also natural applications to comparative genomics, as well as to the emerging field of *comparative metagenomics* that is becoming increasingly crucial for managing and understanding collections of hundreds of thousands of samples, like those already available in [4, 5]. For example, a cluster corresponding to an unknown taxonomic unit could be positioned inside a taxonomy of known genomes by comparing their substring composition, and two metagenomic samples with annotated clusters could be compared in time proportional to the number of clusters, for example using the measures described in [6], rather than in time proportional to the number of distinct substrings of a specific length, as done e.g. in [7, 8], or to the number of reads, as done in [9].

The problem of building a scalable, accurate and unsupervised metagenomic clustering pipeline is fairly recent and still open. Most existing approaches exploit the same signal as alignment-free genome comparison tools, namely taxon-specific biases in the frequency of substrings of a specific length, and they use such signal in hidden Markov models [10, 11], maximum likelihood Monte Carlo Markov chains [12], and expectation maximization algorithms [11, 13]. Other pipelines blend statistics with combinatorial criteria, merging e.g. reads that share long substrings first, and then further merging such groups by similarity of their *k*-mer composition vectors (see e.g. [14–16] and references therein). A single metagenomic sample contains tens of gigabases, and to improve accuracy it is becoming more common to cluster *the union of multiple samples* that are believed to contain shared taxonomic units, as described e.g. in [17, 18]. Notwithstanding such issues of scale, no existing clustering pipeline is designed to be space-efficient, and can thus handle with commodity hardware the largest datasets available.

### Read clustering

Read clustering tools share a number of core combinatorial primitives, which they blend with statistical considerations and with ad hoc heuristics to achieve accuracy. A pipeline that contains most such primitives is the one described in [15], which we summarize in what follows, pointing the reader to the original paper and to its references for statistical considerations and for criteria used to set the parameters. The same primitives recur in a number of other pipelines [14, 16, 19]. As customary, we call *k*-*mer* any string of length *k*, and we call *coverage* the average number of reads that contain a position of the genome of a taxonomic unit. We also use the term *taxon* as a synonym for taxonomic unit.

The first step of the pipeline consists in detecting and filtering out reads sampled from low-frequency taxa, since in practice the presence of such reads tends to degrade the quality of the clusters of high-frequency taxonomic units. Reads from low-frequency taxa should also be clustered with dedicated settings of the parameters. Such filtering has the additional advantage of removing reads with a large number of sequencing errors, and of reducing the size of the input to the following stages. Specifically, given integers *k* and *τ*>1, a read is filtered out iff all its distinct *k*-mers occur (possibly reverse-complemented) less than *τ* times in the read set, where *τ* is set according to error rate, expected coverage, and read length.

Given a DNA string *r*, let $\widetilde {r}$ denote its reverse complement. Consider the graph where reads are vertices and two vertices are connected by an edge iff they are related in the following sense:

#### **Definition 1**

(*k*-RC-relation) Two DNA strings *r*_1_ and *r*_2_ are related iff there is a string *α* of length *k* such that *α* occurs in *r*_1_, and *α* or $\widetilde {\alpha }$ occurs in *r*_2_.

The requirement of sharing a *k*-mer in either the forward or the reverse-complement orientation comes from the fact that we ignore whether a read was sampled from the forward or the reverse-complement strand of its genome. The second step of the pipeline consists in computing the connected components of the read graph defined by the *k*-RC-relation: we call such components the *k-RC connected components*. We omit *k* whenever its value is clear from the context. The value of *k* is typically set using statistics on the substrings of known genomes. The connected components of the *k*-RC-relation loosely correspond to unassembled contigs. Moreover, assuming that the genomes of distinct taxonomic units have approximately the same length, different taxonomic units should get approximately the same number of connected components. Connected components can be further merged if we have paired-end labels on the reads.

In this paper we will also consider the following relation, whose connected components are a refinement of those induced by the previous one:

#### **Definition 2**

(*k*-relation) Two strings *r*_1_ and *r*_2_ are related iff there is a string *α* of length *k* such that *α* occurs in *r*_1_ and *α* occurs in *r*_2_.

Note that there is a one-to-one correspondence between the connected components of the *k*-relation and the connected components of the de Bruijn graph of order *k* of the set of reads.

The third step of the pipeline consists in computing *composition vectors* of *h*-mers, where *h*<*k*, for every connected component: a composition vector is an array of 4^*h*^ elements, where each element corresponds to a distinct *h*-mer, and where the value of element *α* is the (normalized) frequency of string *α* in the connected component. Since reads can be sampled from both strands of a double-stranded DNA molecule, the frequency of an *h*-mer and of its reverse complement are summed, and a composition vector consists just of the distinct *h*-mers that are lexicographically smaller than their reverse complement.

Composition vectors are computed from connected components, rather than from single reads, since reads are typically too short for their *h*-mer composition to approximate the one of their corresponding genomes. Due to multiple occurrences of the same string inside the same connected component, the *h*-mer composition is not estimated directly from the reads in the connected component, but rather from distinct long substrings that repeat inside the connected component, specifically from distinct substrings of length at least *e*>*h* which occur (possibly reverse-complemented) at least *τ* ^′^ times in the connected component.

Composition vectors are finally clustered using e.g. the *k*-means algorithm, since connected components with similar *h*-mer composition are likely to correspond to long fragments of the same genome.

### Strings and string indexes

Let *S*[1,*n*] be a string with alphabet *Σ*=[1,*σ*]. For simplicity, we assume in what follows that *S*[0] means *S*[*n*], and that the substring *S*[0,*n*] means *S*[*n*,*n*]. We denote by $\overline {S}$ the reverse of *S*. Given a bijective mapping *f*:*Σ*→*Σ* that defines a *complement* character for each character in *Σ*, we call *reverse complement* of *S* the string $\widetilde {S} = f(S[n]) \cdot f(S[n-1]) \cdot \cdots \cdot f(S[1])$. Unless otherwise noted, in this paper we assume *f* to be the natural complementarity of DNA bases, i.e. *f*(A)=T, *f*(T)=A, *f*(C)=G, *f*(G)=C, although our algorithms do not exploit this specific mapping.

We denote by $\mathcal {P}_{S}(\alpha)$ the set of all starting positions of a string *α*∈*Σ* ^+^ in the circular version of *S*. We set $\Sigma ^{r}_{S}(\alpha)=\{c \in \Sigma : |\mathcal {P}_{S}(\alpha c)|>0 \}$ and $\Sigma ^{\ell }_{S}(\alpha)=\{c \in \Sigma : |\mathcal {P}_{S}(c \alpha)|>0 \}$. A *repeat* *α*∈*Σ* ^+^ is a string that satisfies $|\mathcal {P}_{S}(\alpha)|>1$. A repeat *α* is *right-maximal* (respectively, *left-maximal*) iff $|\Sigma ^{r}_{S}(\alpha)|>1$ (respectively, iff $|\Sigma ^{\ell }_{S}(\alpha)|>1$). A *maximal repeat* is a repeat that is both left- and right-maximal. It is well known that a maximal repeat corresponds to an equivalence class of the set of all right-maximal repeats. A *supermaximal repeat* is a maximal repeat that is not a substring of any other maximal repeat. We say that a left-maximal repeat *α* is *strongly left-maximal* iff there are at least two distinct characters *a* and *b* in *Σ* such that both *a* *α* and *b* *α* are right-maximal repeats of *S*. Clearly only right-maximal repeats of *S* can be strongly left-maximal, thus the set of strongly left-maximal repeats of *S* is a subset of the maximal repeats of *S*. Given a string *α* that occurs in *S*, we call *λ*(*α*) the number of (not necessarily proper) suffixes of *α* that are strongly left-maximal repeats of *S*, and we call *λ*_*S*_= max{*λ*(*S*[1,*i*]):1≤*i*≤*n*)}. Clearly *λ*_*S*_∈*O*(*n*). Strong right-maximality is defined symmetrically. A string *α*∈*Σ* ^+^ is a *reverse-complement right-maximal repeat* (*RC right-maximal* for short) of *S* if it is a right-maximal repeat of $S\$\widetilde {S}$, where *$* *Σ* is a separator: in other words, there are two distinct characters *c*,*d*∈*Σ* such that *α* *c* or $\widetilde {\alpha c}$ is a substring of *S*, and *α* *d* or $\widetilde {\alpha d}$ is a substring of *S*. Reverse-complement left-maximal repeats and reverse-complement maximal repeats are defined symmetrically.

The *suffix tree* of a string *S*∈[1,*σ*]^+^ is the compacted trie built on the set of all suffixes of string *S* *$*, where *$*=0[1,*σ*] [20]. There is a bijection between the set of leaves of the suffix tree and the set of suffixes of *S* *$*, and there is a bijection between the set of internal nodes of the suffix tree and the set of right-maximal repeats of *S*. We denote by *ℓ*(*v*) the label of a node *v* in the tree, i.e. the concatenation of the labels of all edges in the path from the root to *v*. The *locus* of a nonempty substring *α* of *S* in the suffix tree of *S* is the node *v* such that *α* is a (not necessarily proper) prefix of *ℓ*(*v*) and *ℓ*(*u*) is a proper prefix of *α*, where *u* is the parent of *v*. A *suffix link* connects the node of the suffix tree that corresponds to a string *α*, to the node of the suffix tree that corresponds to the string *α*[2,|*α*|]. Inverting the direction of all suffix links gives the so-called *explicit Weiner links*. The *suffix link tree* of *S* is the trie whose set of nodes consists of the set of all internal nodes of the suffix tree of *S*, and whose set of edges consists of all the explicit Weiner links (that start from internal nodes) of the suffix tree of *S*. An internal node of the suffix tree that corresponds to a right-maximal string *α* is the source of an *implicit Weiner link*, labelled by character *c*, if string *c* *α* occurs in *S*, but is not right-maximal: the target of such implicit Weiner link is the node that corresponds to the shortest string prefixed by *c* *α* that labels a node of the suffix tree. The number of implicit and explicit Weiner links (that start from internal nodes) in the suffix tree of a string *S* *$* of length *n* is upper-bounded by 2*n*−2 [21]. The *generalized suffix tree* of a set of strings *S* ^1^,*S* ^2^,…,*S* ^*m*^ on alphabet [1,*σ*] is the suffix tree of the concatenation *S* ^1^·*$*_1_·*S* ^2^·*$*_2_·⋯·*S* ^*m*^·*$*_*m*_, where *$*_1_,…,*$*_*m*_ are distinct separators that are lexicographically smaller than every character in [1,*σ*].

The Burrows-Wheeler transform (BWT) is a standard tool in text indexing. For convenience, we define the Burrows-Wheeler transform only for strings terminated with a unique character *$*=0 that is lexicographically smaller than all characters in *Σ*. The suffix array *SA*_*S*_[1,*n*] of *S* is an array such that *SA*_*S*_[*i*] is the starting position of the suffix of *S* with lexicographic rank *i* among all suffixes of *S*. The Burrows-Wheeler transform *BWT*_*S*_[1,*n*] of *S* is the string such that *BWT*_*S*_[*i*]=*S*[*SA*_*S*_[*i*]−1] if *SA*_*S*_[*i*]≠1, and *BWT*_*S*_[*i*]=*S*[*n*] otherwise. Given a collection of strings *S* ^1^,*S* ^2^,…,*S* ^*m*^, where *S* ^*i*^∈*Σ* ^+^ for all *i*∈[1,*m*], we call *BWT of the collection* the string *BWT*_*S*_, where *S*=*S* ^1^·*$*_1_·*S* ^2^·*$*_2_·⋯·*S* ^*m*^·*$*_*m*_, and *$*_1_,…,*$*_*m*_ are distinct separators that are lexicographically smaller than every character in *Σ*. The BWT can be used as a full-text index, by encoding it to answer *rank queries* rank _*BWT*_(*i*,*c*), which return the number of times character *c*∈*Σ* occurs in the prefix *BWT*[1,*i*], and by augmenting it with array *C*[1,*σ*], such that *C*[*i*] is the number of characters in *BWT* whose lexicographical rank is strictly less than *i*. In this paper we assume that the BWT is encoded as a wavelet tree, thus rank operations on the BWT take *O*(log*σ*) time [22]. Rank operations on a bitvector of length *n* take constant time if such bitvector is augmented with suitable data structures of *o*(*n*) bits; such data structures can be built in *O*(*n*) time and *o*(*n*) bits of working space [23, 24]. Rank queries and the *C* array enable a *backward step* operation on the BWT: given the lexicographic rank *i* ^′^ of suffix *S*[*i*,*n*], a backward step gives the lexicographical rank of suffix *S*[*i*−1,*n*] using the formula *C*[*BWT*_*S*_[*i* ^′^]]+rank _*BWT*_(*i* ^′^,*BWT*_*S*_[*i* ^′^]). In what follows, we drop the subscript from *SA*, *BWT* and rank whenever it is clear from the context.

We can associate to each substring *α* of *S* the interval *SA*_*S*_[*i*,*j*] that contains the starting positions of all the suffixes of *S* prefixed by *α*, i.e. the starting positions of all occurrences of *α* in *S*. There is a bijection between the set of all such intervals of size at least two and the set of all internal nodes of the suffix tree of *S*. Given any such interval associated with string *α*, and given a character *c*, we can compute the interval of string *c* *α* if it exists (or return an empty interval otherwise), using just two rank queries on *BWT*_*S*_^1^. If *α* is right-maximal, this operation corresponds to taking a Weiner link labelled by *c* from the internal node of the suffix tree labelled by *α*. We can traverse the entire suffix-link tree by performing a linear number of such operations, and by using a suitably designed stack [25]: many algorithms based on the suffix tree can be simulated space-efficiently using such traversal [25].

The *bidirectional Burrows-Wheeler index* [26–29] consists of *BWT*_*S*_ and of $\mathsf {BWT}_{\overline {S}}$, which we also denote by $\overline {\mathsf {BWT}}_{S}$. *BWT* can be interpreted as the list of left extensions of all lexicographically sorted suffixes of *S*, and $\overline {\mathsf {BWT}}$ can be interpreted as the list of right extensions of all *colexicographically* sorted prefixes, where a string *α* is *colexicographically smaller* than a string *β* iff $\overline {\alpha }$ is lexicographically smaller than $\overline {\beta }$. A substring *α* of *S* is associated with a contiguous lexicographic (respectively, colexicographic) interval, i.e. with the lexicographic (respectively, colexicographic) range of all suffixes (respectively, prefixes) of *S* that are prefixed (respectively, suffixed) by *α*. We denote the first and last position of the lexicographic interval of a substring *α* with $i^{\rightarrow }_{\alpha }$ and $j^{\rightarrow }_{\alpha }$, respectively, and the first and last positions of the colexicographic interval of the same substring with $i^{\leftarrow }_{\alpha }$ and $j^{\leftarrow }_{\alpha }$, respectively. Given a string *α*, indices $i^{\rightarrow }_{\alpha }$, $j^{\rightarrow }_{\alpha }$, $i^{\leftarrow }_{\alpha }$, $j^{\leftarrow }_{\alpha }$ and a character *c*∈*Σ*, it is possible to compute a *left-extension*, i.e. the indices $i^{\rightarrow }_{c \alpha }$, $j^{\rightarrow }_{c \alpha }$, $i^{\leftarrow }_{c \alpha }$, $j^{\leftarrow }_{c \alpha }$ and a *right-extension*, i.e. the indices $i^{\rightarrow }_{\alpha c}$, $j^{\rightarrow }_{\alpha c}$, $i^{\leftarrow }_{\alpha c}$, $j^{\leftarrow }_{\alpha c}$ in time *O*(*σ* log*σ*): see Algorithm 1. Within the same space budget, the time complexity can be further improved to *O*(log*σ*), by replacing the sum in line 4 of Algorithm 1 with the count operation provided by wavelet trees [30], and finally to *O*(1) by using monotone minimal perfect hash functions [25]. In what follows, we use extendLeft and extendRight to denote these two primitives of a bidirectional BWT index. We also assume that a bidirectional BWT index provides operation enumerateLeft (respectively, enumerateRight), which, given a string *α*, $i^{\rightarrow }_{\alpha }$, $j^{\rightarrow }_{\alpha }$ (respectively, $i^{\leftarrow }_{\alpha }$, $j^{\leftarrow }_{\alpha }$), and a character *c*∈*Σ*, returns the set of all *d* distinct characters that occur in $\mathsf {BWT}_{S}[i^{\rightarrow }_{\alpha }, j^{\rightarrow }_{\alpha }]$ (respectively, in $\overline {\mathsf {BWT}}_{S}[i^{\leftarrow }_{\alpha }, j^{\leftarrow }_{\alpha }]$), in lexicographic order. Operations enumerateLeft and enumerateRight can be implemented in *O*(*d* log(*σ*/*d*)) time using wavelet trees [28].



## Methods

We show how to implement in small space the key primitives of the read clustering pipeline, using the bidirectional BWT index of the concatenation of all reads in the sample. Specifically, we focus on the step that builds the connected components, since this is the space bottleneck of the entire pipeline in practice, and since the same techniques can be applied to the initial filtering of reads from low-frequency taxa. Building composition vectors and clustering them requires negligible space compared to the other steps.

We say that the *rank of a read* is the number of reads that come before it in the concatenation, plus one to make the ranks start from one. We first describe how to iterate over all the RC right-maximal substrings of *S*, a result that will be useful in what follows:

### **Lemma 1**

Given the bidirectional BWT index of a string *S* ∈[1,*σ*]^*n*−1^ *$*, where *$*=0, we can iterate over all the RC right-maximal substrings of *S* in *O*(*n* log*σ*) time and *O*(*σ* log2*n*) bits of space in addition to the input and the output.

### *Proof*

We use the recursive procedure in Algorithm 2 to enumerate all the nodes of the generalized suffix tree of *S* and $\widetilde {S}$, as described in [25]. Each frame in the iteration stack represents the four intervals that identify the lexicographic and colexicographic ranges of a string and its reverse complement. To decide whether substring *c* *α* is RC right-maximal, we just need intervals $[i^{\leftarrow }_{c \alpha }, j^{\leftarrow }_{c \alpha }] $ and $[i^{\rightarrow }_{\widetilde {{c \alpha }}}, j^{\rightarrow }_{\widetilde {{c \alpha }}}] $. Recall that interval $ [i^{\leftarrow }_{c \alpha }, j^{\leftarrow }_{c \alpha }] $ in the reverse BWT lists all the right extensions of *c* *α*, and interval $[i^{\rightarrow }_{\widetilde {{c \alpha }}}, j^{\rightarrow }_{\widetilde {{c \alpha }}}]$ in the forward BWT lists all the left extensions of $\widetilde {c \alpha }$. Let $\Sigma ^{\prime }_{1} = \{c : c \in \overline {\mathsf {BWT}}[i^{\leftarrow }_{c \alpha },j^{\leftarrow }_{c \alpha }]\}$ be the set of distinct characters in $\overline {\mathsf {BWT}}[i^{\leftarrow }_{c \alpha },j^{\leftarrow }_{c \alpha }]$, and let $\Sigma '_{2} = \{\widetilde {c} : c \in \mathsf {BWT}[i^{\rightarrow }_{\widetilde {{c \alpha }}}, j^{\rightarrow }_{\widetilde {{c \alpha }}}]\}$ be the set of distinct *reverse complements* of the characters in $\mathsf {BWT}[i^{\rightarrow }_{\widetilde {{c \alpha }}}, j^{\rightarrow }_{\widetilde {{c \alpha }}}]$. String *c* *α* is RC right-maximal iff $\left | \Sigma ^{\prime }_{1} \cup \Sigma ^{\prime }_{2} \right |>1$: this can be checked by calling the enumerateLeft and enumerateRight operations provided by the bidirectional index, and by taking the union of their output.

Every element in the output of enumerateLeft and enumerateRight can be either charged to an implicit or explicit Weiner link of the generalized suffix tree of *S* and $\widetilde {S}$, or to an edge of the same tree, thus the total number of such calls is *O*(*n*), and the total number of calls to extendLeft and extendRight is *O*(*n*) as well. The claimed time bound comes from properties described in the “Background” section. As used in Algorithm 2, the stack takes *O*(*λ*_*S*_ *σ* log*n*) bits, since in the worst case it consists of *λ*_*S*_ levels, each of which contains up to *σ* quadruplets of intervals in *BWT* and $\overline {\mathsf {BWT}}$. We reduce the number of levels in the stack to *O*(log*n*) by pushing first, at every iteration, the left-extension with largest sum of interval lengths in, say, *BWT*, as described in [25]. □



Recall that in our case *S* is a collection of reads, thus, even without applying the logarithmic stack technique described in [25], the space used by Lemma 1 is *O*(*ℓ* *σ* log*n*) bits, where *ℓ* is the maximum length of a read.

We compute *k*-RC connected components in two steps: first, we compute connected components of the *k*-relation on reads. Then, we merge every two connected components *C*_1_ and *C*_2_ for which there is a *k*-mer *α* such that *α* is contained in some read in *C*_1_, and $\widetilde {\alpha }$ is contained in some read in *C*_2_. As done in [15], we use a union-find data structure on the set of reads to implement the merging operations (see e.g. [31]). We assume that such data structure supports the following queries: find(*r*), which returns the handle of the connected component containing read *r*; union(*C*_1_,*C*_2_), which merges components *C*_1_ and *C*_2_ and returns the handle of the resulting component; and size(*C*), which returns the number of reads in component *C*. We initialize the data structure so that every read belongs to a distinct component.

### **Lemma 2**

Let *S*=*S* ^1^ *$* *S* ^2^ *$*⋯*S* ^*m*^ *$* *$* be a string of length *n* such that *S* ^*i*^∈*Σ* ^+^ for all *i*∈[1,*m*], and *$*=0 is a separator. Assume that we are given the bidirectional BWT index of *S*, a union-find data structure initialized with *m* sets and supporting find and union in time *t*, and an integer *k*. Then we can encode, in the union-find data structure, all the connected components of the *k*-relation graph on set {*S* ^1^,*S* ^2^,…,*S* ^*m*^}, in *O*(*n*(*t*+ log*σ* ^′^)) time and in *n*+*o*(*n*)+*O*(max{*ℓ* *σ* ^′^ log*n*,*K* log*m*}) bits of space in addition to the input, where *σ* ^′^=*σ*+1, *ℓ*= max{|*S* ^*i*^|:*i*∈[1,*m*]} and *K* is the number of distinct *k*-mers of *S*.

### *Proof*

We enumerate the nodes of the suffix tree of *S* in the order induced by the suffix-link tree of *S*, using a recursive procedure similar to Algorithm 2. Specifically, we keep just $[i^{\rightarrow }_{\alpha },j^{\rightarrow }_{\alpha }]$ and $[i^{\leftarrow }_{\alpha },j^{\leftarrow }_{\alpha }]$ for every right-maximal substring *α* of *S*, and we use the fact that *α* is right-maximal iff $\overline {\mathsf {BWT}}[i^{\leftarrow }_{\alpha },j^{\leftarrow }_{\alpha }]$ contains at least two distinct characters (see [25] for further details). Note that the *BWT* intervals of distinct *k*-mers are disjoint. Thus, during the iteration, we mark in a bitvector *B*, of length equal to the size of *BWT*, the first position of the lexicographic interval of every *k*-mer. This can be done as follows (see e.g. [21]). We initialize *B* to all ones and, whenever we enumerate a right-maximal substring *α* of length at least *k*, we use operations enumerateRight and extendRight provided by the bidirectional index to compute the interval $[i^{\rightarrow }_{\alpha c},j^{\rightarrow }_{\alpha c}]$ of every right-extension *α* *c* of *α*, in lexicographic order. Then, we flip bit $B[i^{\rightarrow }_{\alpha c}]$ for all *c* except the first in lexicographic order. At the end of this process we index *B* to answer rank queries in constant time, so that we can compute the ID of the *k*-mer whose *BWT* interval contains a given position *i* in *BWT* by rank _*B*_(*i*).

Every *k*-mer interval is associated with the set of distinct reads that contain the starting points of the suffixes of *S* inside the interval. For each *k*-mer interval, we store the handle of one of such read. The handles are stored in an array *H*, of length equal to the number of *k*-mer intervals, such that the handle corresponding to the interval of the *k*-mer that contains position *i* in *BWT* is stored in *H*[rank _*B*_(*i*)]. We initialize array *H* with null values. Then, we backward-search string *S* in *BWT*_*S*_, maintaining the lexicographic rank *i* ^→^ of the suffix that starts at the current position, and the rank *r* of the read that contains such suffix. At each step we compute *p*, the ID of the *k*-mer whose interval contains *i* ^→^: if *H*[*p*] is null, we set *H*[*p*] to find(*r*); otherwise, if *H*[*p*] is different from find(*r*), we set *H*[*p*] to the output of union(*H*[*p*],find(*r*)). □

Note that in Lemma 2 we do not use a distinct separator for every read, but instead we use the same separator for all reads. The result is unaffected by this change, and we will use this convention in the rest of the paper. We leave details to the reader.

Clearly it suffices to consider just *k*-mers that do not contain *$* and that occur at least twice in *S*. More tightly, it suffices to consider just right-maximal *k*-mers that do not contain *$*: indeed, if a *k*-mer *α* is always followed in *S* by character *c*, then the set of reads that are merged by *α* is a subset of the set of reads that are merged by *α*[2,*k*]·*c*. Lemma 2 can be adapted to use just right-maximal *k*-mers:

### **Corollary 1**

Lemma 2 can be implemented in *n*+*o*(*n*)+*O*(max{*ℓ* *σ* ^′^ log*n*,*K* ^′^ log*m*}) bits of space in addition to the input, where *K* ^′^ is the number of distinct right-maximal *k*-mers of *S*.

### *Proof*

We follow the same approach as in Lemma 2. The intervals of all right-maximal *k*-mers are disjoint and of size at least two. We mark the first and the last position of every such interval in array *B*, by iterating over all right-maximal substrings of *S* and by setting $B[i^{\rightarrow }_{\alpha }]=1$ and $B[j^{\rightarrow }_{\alpha }]=1$ for every right-maximal *α* of length *k*. This marking technique was introduced independently by [21, Lemma 16.5] and by [32]. As we backward-search *S* in *BWT*_*S*_, we decide whether the *k*-mer that prefixes the current suffix of *S* is right-maximal, by checking whether *B*[*i*]=1 or rank _*B*_(*i*) is odd, where *i* is the lexicographic rank of the current suffix. We proceed only in the positive case, using the handle that corresponds to the *k*-mer located at position ⌈rank _*B*_(*i*)/2⌉ of *H*. □

Note that the running time of Corollary 1 is *O*(occ·*t*+*n* log*σ*), where occ is the total number of occurrences of all right-maximal *k*-mers. In real datasets, for typical values of *k* (e.g. 36), the number of distinct *k*-mers can be approximately 45 times bigger than the number of distinct right-maximal *k*-mers, and the length of the string can be approximately 17 times bigger than the number of occurrences of right-maximal *k*-mers.

Consider the set $\mathcal {R}$ of all distinct maximal repeats of *S* of length at least *k*: every substring *S*[*i*,*j*] that equals a right-maximal *k*-mer of *S* is a suffix of a substring *S*[*i* ^′^,*j*], with *i* ^′^≤*i*, that equals a maximal repeat in $\mathcal {R}$, and every substring *S*[*i* ^′^,*j*] that equals a maximal repeat in $\mathcal {R}$ has a right-maximal *k*-mer as a suffix. Thus, issuing union queries using the elements of $\mathcal {R}$ is equivalent to issuing union queries using all right-maximal *k*-mers. The size of $\mathcal {R}$, however, is *at least* the number of right-maximal *k*-mers. Specifically, the number of right-maximal *k*-mers equals the size of set $\mathcal {R}^{\prime } \subseteq \mathcal {R}$, where $\mathcal {R}'$ is the set of elements of $\mathcal {R}$ that do not have another element of $\mathcal {R}$ as a suffix. In other words, the elements of $\mathcal {R}^{\prime }$ are the reversed labels of the loci of the reversed right-maximal *k*-mers of *S* in the suffix tree *of the reverse of S*.

More tightly, every substring *α* of a maximal repeat $\beta \in \mathcal {R}$ occurs in *S* whenever *β* occurs, and possibly at other positions, therefore the union operations induced by *β* are a subset of the union operations induced by *α*, and we can safely disregard *β* for clustering. We are thus interested in the following subset of the maximal repeats of *S*:

### **Definition 3**

Let *S*∈*Σ* ^*n*^ be a string and let *k* be an integer. A repeat of *S* is called *k-submaximal* if it is a maximal repeat of *S* of length at least *k*, and if it does not contain any maximal repeat of length at least *k* as a substring.

Note that the set of *k*-submaximal repeats is a subset of $\mathcal {R}^{\prime }$. Lemma 2 can be adapted to use just the *k*-submaximal repeats of *S*:

### **Corollary 2**

Lemma 2 can be implemented in 2*n*+*o*(*n*)+*O*(max{*ℓ* *σ* ^′^ log*n*,*K* ^′′^ log*m*}) bits of space in addition to the input, where *K* ^′′^ is the number of distinct *k*-submaximal repeats of *S*.

### *Proof*

Since the set of all *k*-submaximal repeats is a subset of $\mathcal {R}^{\prime }$, and since the elements of $\mathcal {R}^{\prime }$ are the reversed labels of the loci of the reversed right-maximal *k*-mers of *S* in the suffix tree of $\overline {S}$, there is a one-to-one correspondence between the set of occurrences of *k*-submaximal repeats and the set of occurrences of their right-maximal suffixes of length *k*. We can thus issue union queries using just right-maximal *k*-mers that are the (not necessarily proper) suffix of a *k*-submaximal repeat, or equivalently using just right-maximal *k*-mers such that the label of the locus of their reverse in the suffix tree of $\overline {S}$ is the reverse of a *k*-submaximal repeat.

Assume that we have bitvector *B* from Corollary 1, with the intervals of all right-maximal *k*-mers marked with ones, indexed to support rank queries. We mark in another bitvector *B* ^′^ (initialized to all zeros) the subset of such intervals that correspond to *k*-mers that are the suffix of a *k*-submaximal repeat, as follows. We scan *B* sequentially, and for every pair (*i*,*j*) of ones such that the first has odd rank *x* and the second has even rank *x*+1, we check whether *BWT*_*S*_[*i*,*j*] contains at least two distinct characters: if so, the right-maximal *k*-mer *α* that corresponds to interval [*i*,*j*] is also left-maximal, *α* is a *k*-submaximal repeat, and we set *B* ^′^[*i*]=*B* ^′^[*j*]=1.

Otherwise, let *v* be the locus of $\overline {\alpha }$ in the suffix tree of $\overline {S}$, let *u* be the parent of *v*, let the label of *v* be *ℓ*(*v*)=*ℓ*(*u*)*β* *γ*, let $\overline {\alpha }=\ell (u)\beta $, and let |*γ*|=*g*. We iteratively take backward steps from [*i*,*j*] until we find a BWT interval that contains at least two distinct characters. This is equivalent to reading the characters of *γ* sequentially. Let such sequence of backward steps yield intervals [*i*_1_,*j*_1_],[*i*_2_,*j*_2_],…,[*i*_*g*_,*j*_*g*_] corresponding to right-maximal strings $\gamma [1]\alpha, \gamma [2]\gamma [1]\alpha, \dots, \overline {\gamma }\alpha $. Assume that, using rank queries on *B*, we detect that one such interval [*i*_*y*_,*j*_*y*_] is contained inside the interval of a right-maximal *k*-mer *θ*. Let *v* ^′^ be the locus of $\overline {\theta }$ in the suffix tree of $\overline {S}$. Then *ℓ*(*v* ^′^) is a substring of *ℓ*(*v*) and $\overline {\ell (v^{\prime })}$ is an element of $\mathcal {R}^{\prime }$, thus $\overline {\ell (v)}$ is not *k*-submaximal, we leave *B* ^′^[*i*] and *B* ^′^[*j*] to zero, and we move to the next pair of ones in *B*. If none of the intervals [*i*_1_,*j*_1_],…,[*i*_*g*_,*j*_*g*_] is contained inside the interval of a right-maximal *k*-mer, we set *B* ^′^[*i*]=*B* ^′^[*j*]=1 and we move to the next pair of ones in *B*.

At the end of this process, we index *B* ^′^ for rank queries, we replace the indexed *B* with the indexed *B* ^′^, and we continue as in Corollary 1. The total number of backward steps performed by the algorithm is *O*(*n*), since every step visits a distinct right-maximal substring of *S*. □

Slightly more involved arguments allow one to shave *n* bits from the space complexity of Corollary 2. The running time of Corollary 2 is *O*(occ·*t*+*n* log*σ*), where occ is the total number of occurrences of all *k*-submaximal repeats. In real datasets, for typical values of *k* (e.g. 36), the number of right-maximal *k*-mers can be approximately 1.8 times the number of *k*-submaximal repeats, and the total number of occurrences of right-maximal *k*-mers can be approximately 1.5 times the number of occurrences of *k*-submaximal repeats. Once again, we can discard *k*-submaximal repeats that contain *$*.

Before completing the construction of the *k*-RC connected components, we note that the technique described in Lemma 2 allows one to detect reads whose *k*-mers occur all less than *τ* times in the dataset (without considering reverse complements), in *O*(*n* log*σ* ^′^) time and in *n*+*O*(*ℓ* *σ* ^′^ log*n*) bits of space in addition to the input and the output. Once all reads from low-frequency species have been detected, it is also possible to derive the BWT of such reads, as well as the BWT of all reads from high-frequency species, directly from *BWT*_*S*_. We leave such details to the reader.

To complete the pipeline, we just need to merge all pairs of components *C*_1_ and *C*_2_ that share a reverse complemented *k*-mer. Once again, it suffices to consider just the RC right-maximal *k*-mers that occur in both *S* and $\widetilde {S}$:

### **Lemma 3**

Let *S*=*S* ^1^ *$* *S* ^2^ *$*⋯*S* ^*m*^ *$* *$* be a string of length *n* such that *S* ^*i*^∈*Σ* ^+^ for all *i*∈[1,*m*], and *$*=0 is a separator. Assume that we are given the bidirectional BWT index of *S*, a union-find data structure initialized with *m* sets and supporting find and union in time *t*, and an integer *k*. Then we can encode, in the union-find data structure, all the connected components of the *k*-RC-relation on set {*S* ^1^,*S* ^2^,…,*S* ^*m*^}, in *O*(*n*(*t*+ log*σ* ^′^)) time and in 2*n*+*o*(*n*)+*O*(max{*ℓ* *σ* ^′^ log*n*,*K* ^′′′^ log*m*}) bits of space in addition to the input, where *σ* ^′^=*σ*+1, *ℓ*= max{|*S* ^*i*^|:*i*∈[1,*m*]} and *K* ^′′′^ is the number of distinct RC right-maximal *k*-mers of *S*.

### *Proof*

Let *B*_2_ and *B*_3_ be two bitvectors, of length equal to the length of *BWT*, initialized to all zeros. We iterate over every RC right-maximal *k*-mer *α* using Algorithm 2: if none of the intervals $[i^{\rightarrow }_{\alpha },j^{\rightarrow }_{\alpha }]$ and $[i^{\rightarrow }_{\widetilde {\alpha }},j^{\rightarrow }_{\widetilde {\alpha }}]$ is empty, then the reads corresponding to interval $[i^{\rightarrow }_{\alpha },j^{\rightarrow }_{\alpha }]$ should be in the same connected component as the reads corresponding to interval $[i^{\rightarrow }_{\widetilde {\alpha }},j^{\rightarrow }_{\widetilde {\alpha }}]$. Thus, if $i^{\rightarrow }_{\alpha } \neq j^{\rightarrow }_{\alpha }$ we set $B_{2}[i^{\rightarrow }_{\alpha }]=1$ and $B_{2}[j^{\rightarrow }_{\alpha }]=1$, otherwise we set $B_{3}[i^{\rightarrow }_{\alpha }]=1$. Similarly, if $i^{\rightarrow }_{\widetilde {\alpha }} \neq j^{\rightarrow }_{\widetilde {\alpha }}$ we set $B_{2}[i^{\rightarrow }_{\widetilde {\alpha }}]=1$ and $B_{2}[j^{\rightarrow }_{\widetilde {\alpha }}]=1$, otherwise we set $B_{3}[i^{\rightarrow }_{\widetilde {\alpha }}]=1$. At the end of this process we index *B*_2_ and *B*_3_ for rank queries, we allocate a vector *H*_2_ of length equal to the number of distinct intervals marked in *B*_2_, and we store in *H*_2_[*i*] the handle of any read that contains the *k*-mer that corresponds to the *i*-th marked interval, by backward-searching *S* in *BWT*_*S*_ as described in Corollary 1. We similarly fill a vector *H*_3_, of length equal to the number of bits marked in *B*_3_. Finally, we use again Algorithm 2 to iterate over every RC right-maximal *k*-mer *α*: if none of the intervals $[i^{\rightarrow }_{\alpha },j^{\rightarrow }_{\alpha }]$ and $[i^{\rightarrow }_{\widetilde {\alpha }},j^{\rightarrow }_{\widetilde {\alpha }}]$ is empty, we issue union(*h*_1_,*h*_2_), where $h_{1} = H_{2}[ \lceil \text {\texttt {rank}}_{B_{2}}(i^{\rightarrow }_{\alpha })/2 \rceil ]$ if $i^{\rightarrow }_{\alpha } \neq j^{\rightarrow }_{\alpha }$, otherwise $h_{1} = H_{3}[ \text {\texttt {rank}}_{B_{3}}(i^{\rightarrow }_{\alpha }) ]$. Similarly, $h_{2} = H_{2}[ \lceil \text {\texttt {rank}}_{B_{2}}(i^{\rightarrow }_{\widetilde {\alpha }})/2 \rceil ]$ if $i^{\rightarrow }_{\widetilde {\alpha }} \neq j^{\rightarrow }_{\widetilde {\alpha }}$, otherwise $h_{2} = H_{3}[ \text {\texttt {rank}}_{B_{3}}(i^{\rightarrow }_{\widetilde {\alpha }}) ]$. □

Note that, if the complementation function reverses the alphabet (and DNA complementation does), we can avoid executing Algorithm 2 twice. Indeed, we could just run Algorithm 2 and mark in a bitvector *A* the interval of *α* in *BWT*, and in a bitvector *B* the interval of $\widetilde {\alpha }$ in $\overline {\mathsf {BWT}}$, for every RC right-maximal *k*-mer *α* that occurs both in *S* and in $\widetilde {S}$. Then, we could allocate two vectors *H*_*a*_ and *H*_*b*_, of length equal to the number of marked intervals in *A* and *B*, and we could store in *H*_*a*_[*i*] (respectively, in *H*_*b*_[*i*]) the handle of any read that contains the *k*-mer corresponding to the *i*-th marked interval in *A* (respectively, in *B*). Finally, we could issue union(*H*_*a*_[*i*],*H*_*b*_[*K* ^′′^−*i*+1]) for all *i*∈[1,*K* ^′′′^].

Recall that the very last step of the pipeline consists in extracting repeated substrings of length at least *e*>*h* from each connected component. Every such string is a substring of a maximal repeat of $S \cdot \widetilde {S}$ of length at least *e*. If the user has set *e*>*k*, every such maximal repeat occurs in exactly one connected component: we could thus extract all the (supermaximal) repeats of $S \cdot \widetilde {S}$ of length at least *e*, in a single traversal of the generalized suffix-link tree of *S* and $\widetilde {S}$ and within the same budget as the other algorithms (see [25] for details).

Finally, the value of *k* in the *k*-RC-relation can be estimated from the dataset itself: specifically, given a range [*k*_*x*_,*k*_*y*_] of possible values, one might want to compute the value of *k* such that the majority of distinct *k*-mers of *S* and $\widetilde {S}$ occur at least twice in $S \cdot \widetilde {S}$, i.e. most of such *k*-mers are not likely to contain sequencing errors. Such *k* can be computed within the same time and space budget as the algorithms in this paper, using the algorithm described in [33].

In practice the memory used by the enumeration stack is negligible in all algorithms, the peak space usage of the entire pipeline is achieved by Lemma 3 and, assuming that the bidirectional index takes 2*n* log*σ* ^′^+*o*(*n* log*σ* ^′^) bits, such peak is approximately 2*n* log*σ* ^′^+(2*m*+*K* ^′′′^) log*m*+2*n*+*o*(*n* log*σ* ^′^) bits. The 2*m* log*m* bits of space come from the union-find data structure, which stores for each component a pointer to its parent in a tree structure, and the size of the subtree attached to it to maintain balancing. Note that 2*m* log*m*∈*O*(*n* log*σ*) if all reads are distinct. Rather than using the union-find data structure for clustering reads, we could use it for clustering distinct *k*-mers or repeats, and then we could propagate such clustering to reads (as done e.g. in [19]). This could decrease peak memory when clustering the union of a large number of very similar samples.

## Results

The purpose of this section is just to show that our algorithms are practical. Our implementation of the bidirectional BWT index is based on C++, on the SDSL library [34], and on the ropebwt2 library [35]. For simplicity we implement Corollary 1 rather than Corollary 2. We use the multithreading support of the C++ 11 standard library to take advantage of multiple cores.

Specifically, since all our algorithms are traversals of a suffix-link tree, we run them on *c* parallel cores by dividing the BWT into *c* intervals of similar length and by assigning each interval to a distinct core. This work balancing technique is effective, since the length of the BWT interval of a node *v* of the suffix tree correlates well in practice with the number of nodes in the subtree of the suffix-link tree rooted at *v*. We parallelize the backward search of a sequence of reads in its own BWT by dividing the sequence into *c* blocks of approximately equal length, and by backward-searching each block in parallel.

We observe that the parallel traversal of the suffix-link tree fails to use more than four cores efficiently, thus more advanced work-balancing strategies might be needed: engineering our implementation to exploit a large number of cores is outside the scope of this paper.

The other purpose of this section is to show the potential of our framework, both in terms of clustering quality and in terms of computational resources, by comparing our implementation (called *bwtCluster*) to a sampler of recent, state-of-the-art tools. Specifically, we compare bwtCluster to MetaCluster [15], MBBC [11] and BiMeta [14]. Such comparisons are inherently unfair, for a number of reasons. First, MetaCluster is a highly engineered, parallel version of the read clustering pipeline, extensively tuned over multiple years both in terms of quality and of speed [15, 36–39]. Comparing bwtCluster to MetaCluster should thus penalize bwtCluster in terms of quality, and possibly of speed. Second, BiMeta and MBBC differ from the pipeline we described, BiMeta is single-threaded, and MBBC uses less than two cores on average, thus they could be penalized in terms of speed. Performing an extensive analysis of the clustering results of our framework, and augmenting it with advanced heuristics to make it as accurate as possible, are outside the scope of this paper.

To the best of our knowledge there is no standard dataset for evaluating the performance of unsupervised metagenomic clustering algorithms yet, thus we experiment with the following samples of increasing complexity. First, we build three simple, error-free datasets, to measure how well an algorithm can separate two species that belong to distinct units at different levels in the taxonomy. Such datasets contain exactly two species each, with tenfold coverage and paired-end reads of length 100 base pairs, with no errors^2^. We call the datasets the species level, genus level and family level datasets, respectively. The reference genomes are taken from the NCBI database, and sampled at random locations of the genomes. Second, we replicate the simulated, high-complexity datasets A, B and C described in [15]. Such datasets have realistic error rates, contain up to a hundred species, and have different fractions of low-abundance species. The datasets are created by feeding the reference genomes from NCBI to the Metasim software by [40]. Third, we pick two real samples: a sample from the human gut catalogue described in [41] containing 1.4 billion base pairs, and a sample from a study on the mouse gut described in [42] containing 830 million base pairs ^3^.

In simulated datasets, we assess the quality of both the *k*-RC connected components and of the clusters produced by *k*-means, using the measures described in [15]. Specifically, suppose there are *N* species in the dataset, and that an algorithm outputs *M* clusters. Let *R*_*ij*_ be the number of reads in cluster *i* that are from species *j*. We call *precision* the ratio between $\sum _{i=1}^{M} \max _{j} R_{ij}$ and the number of reads in all clusters, and we call *sensitivity* the ratio between $\sum _{j=1}^{N} \max _{i} R_{ij}$ and the total number of reads in dataset. For brevity we call *preclusters* the *k*-RC connected components in what follows. We combine the preclusters and the final clusters produced by both rounds of MetaCluster in order to compute precision and sensitivity. We do not measure clustering quality in real samples, since the truth is not known.

We tried to make our tool as close as possible to MetaCluster by implementing many heuristics found in the MetaCluster papers and even by looking at the source code of MetaCluster, and implementing details not present in the Metacluster papers. Specifically, we do not merge a pair of connected components if either of the components has at least 1000 reads, unless one of the components has size less than 100, and before running *k*-means we filter all connected components containing less than 200 reads. We set the parameters as recommended in [15], namely we set *k*=16 and *τ*=4 for filtering, we set *k*=36 for clustering, and we use *k*-mers of length 5 in the composition vectors clustered by *k*-means. Other MetaCluster heuristics that are not yet implemented in our tool include issuing union queries in increasing order of *k*-mer frequency, merging two reads if they contain two *k*-mers at edit distance one from each other, and a few additional heuristics for growing the sizes of the *k*-RC connected components. Unlike MetaCluster, our tool runs only a single clustering round, but since the number of filtered reads is small, the effect of this is negligible in final the precision and sensitivity.

We ran the four tools for a maximum of 24 hours on each dataset. The results are shown in Table 1.^4^ The tools bwtCluster and BiMeta cannot estimate the number of species in a sample, so we gave the true number of species as parameters to all tools, and we set the number of species to 100 for the real samples. MBBC takes in input an initial guess on the number of species. For the species, genus and family level datasets, when MBBC was given the true number of species as the initial guess, it failed and predicted just one species. With the initial guess of 10 the tool predicted the correct number of species for those datasets, and the numbers reported in Table 1 for such datasets are with the initial guess of 10. For the datasets A, B and C, we gave MBBC the true number of species.

**Table 1:** Precision, sensitivity, peak memory usage and wall clock time of the following clustering algorithms: bwtCluster (BWT), MetaCluster (MC), MBBC, and BiMeta (BM)

Dataset	Size	Tool	Precluster		Cluster		Mem.	Time
			prec.	sens.	prec.	sens.	(GB)	
Species level	0.1	BWT	0.84	0.18	0.83	0.8	0.3	3.7 m
		MC	×	×	×	×	×	×
		MBBC			0.7	0.7	3.8	7.3 m
		BM			0.52	0.76	2.6	9.1 m
Genus level	0.1	BWT	0.87	0.09	0.87	0.84	0.3	3.7 m
		MC	×	×	×	×	×	×
		MBBC			0.79	0.79	3.9	8.7 m
		BM			0.59	0.59	2.5	9.1 m
Family level	0.1	BWT	0.94	0.12	0.94	0.91	0.3	3.9 m
		MC	×	×	×	×	×	×
		MBBC			0.78	0.78	3.8	4.6 m
		BM			0.65	0.65	2.6	9 m
A	1.7	BWT	0.84	0.01	0.71	0.34	6.4	1 h
		MC	0.90	0.05	0.76	0.71	22.4	41 m
		MBBC					≥64	≥24 h
		BiMeta					≥69	≥24 h
B	0.6	BWT	0.92	0.01	0.76	0.72	1.9	27 m
		MC	0.97	0.05	0.82	0.37	10.5	11 m
		MBBC					≥27	≥24 h
		BM			0.30	0.52	22	4.6 h
C	1.7	BWT	0.84	0.01	0.69	0.40	5.7	1 h
		MC	0.90	0.05	0.71	0.70	22.3	43 m
		MBBC					≥65	≥24 h
		BM					≥59	≥24 h
Human gut	1.4	BWT					4.1	53 m
		MC					×	×
		MBBC					≥35	≥24 h
		BM					≥17	≥24 h
Mouse gut	0.8	BWT					2.2	25 m
		MC					×	×
		MBBC					≥22	≥24 h
		BM					≥35	≥24 h

The size of each dataset is given in billion base pairs (Gbp). Algorithms that return an error are marked with symbol ×

**Fig. 1:**
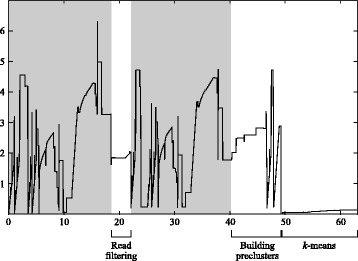
Running bwtCluster on dataset A: time (*horizontal axis*, minutes) versus memory (*vertical axis*, gigabytes). BWT index constructions are highlighted in *gray*

Our tool was the only one which was able to process each dataset within 24 hours without returning an error. The peak memory of our tool was between 3.5 to 14.2 smaller then the competing tools. On the species, genus and family-level datasets, as well as on both real datasets, MetaCluster halted with an error, before even running *k*-means, saying that the number of clusters was too low, due to low coverage. The same error persisted when we tried to run just the second phase, which is designed to cluster low-frequency species. The peak memory usage of bwtCluster was less than 4 bytes per character (Table 1) and it occurred during the construction of the index (Fig. 1), thus it might be further reduced by replacing the BWT construction library. We could also be more careful in keeping in memory just the data structures that are strictly necessary to each step of the pipeline.

On datasets A, B and C, bwtCluster had approximately 94% of the precision of MetaCluster, both in the final clusters and in the preclusters, suggesting that our clusters are approximately as clean as MetaCluster’s. The precluster sensitivity of bwtCluster, however, was just approximately 20% of the precluster sensitivity of MetaCluster, suggesting that bwtCluster fragments species into more preclusters than MetaCluster: this could be caused e.g. by the absence of approximate matching and of other advanced merging heuristics implemented in MetaCluster. Both MBBC and BiMeta generally had smaller precision and sensitivity compared to bwtCluster.

In conclusion, every competing tool we considered is either unstable, or it is significantly slower than our implementation, or it uses significantly more memory, and no competitor with a stable implementation achieves higher precision or sensitivity than bwtCluster on a substantial number of datasets. Finally we note that the implementation of MetaCluster requires that all reads in a sample are of equal length, and have length at most 128 base pairs, whereas our tool has no such restriction.

## Discussion and conclusions

We described an algorithmic framework for unsupervised read clustering in small space, based on the bidirectional Burrows-Wheeler index of a metagenomic sample. Specifically, we identified a set of core combinatorial primitives and we implemented them in *O*(*n*(*t*+ log*σ*)) time using 2*n*+*o*(*n*)+*O*(max{*ℓ* *σ* log*n*,*K* log*m*}) bits of space in addition to the index and to a union-find data structure on the set of reads, where *n* is the total number of characters in the sample, *m* is the number of reads, *σ* is the total size of the alphabet, *t* is the query time of the union-find data structure, and *K* is a measure of the redundancy of the sample, like the number of distinct right-maximal substrings of fixed length *k*, or the number of distinct submaximal repeats of length at least *k*. In practice both *σ* and *t* are constant, since *t* can be for example *O*(*x*), where *x* is the value such that the Ackermann function *A*(*x*,*x*) equals *m* [31]. Our algorithms are practical, and they can exploit multiple cores by a parallel traversal of the suffix-link tree of the sample.

Since our algorithms use a string index as their substrate, one can build such index just once, and run the algorithms multiple times with different settings of the parameters. Approximately half of the time taken by our implementation is spent in building the index (Fig. 1), thus building the index just once is likely to speed up this frequent use case in explorative data analysis. Since the index is based on the ubiquitous Burrows-Wheeler transform, such transform might have already been computed for supporting other queries, making such algorithms immediately applicable to existing datasets.

Compressed representations of the BWT could reduce peak space even further. Specifically, the BWT of the union of similar metagenomic samples is likely to be very compressible, and since the space used by our algorithms in addition to the BWT is dominated by a measure of the redundancy of the input, such space is not likely to grow significantly when multiple similar samples are clustered at the same time.

Finally, one could experiment with dropping the *k*-RC-relation altogether, and with merging reads using just the *k*-relation: a connected component would then correspond to a substring of a genome *in a specific orientation*, and two connected components that originate from reading the same substring in different orientations would likely be merged during the final *k*-means step, since their composition vectors are similar. This would remove the need for storing $\overline {\mathsf {BWT}}_{S}$ in all steps after the initial filtering of reads from low-frequency taxa, since the corresponding algorithms can be implemented on top of the unidirectional traversal described in [43].

## Endnotes

^1^More precisely if the interval of *α* is [*i*,*j*] then the interval of *c* *α* will be [*i* ^′^,*j* ^′^], where *i* ^′^=*C*[*c*]+rank _*BWT*_(*i*−1,*c*)+1 and *j* ^′^=*C*[*c*]+rank _*BWT*_(*j*,*c*).

^2^The first dataset contains species *Vibrio cholerae* and *Vibrio vulnificus*, the second *Vibrio cholerae* and *Photobacterium gaetbulicola*, the third *Vibrio cholerae* and *Escherichia coli*

^3^EBI identifier SAMEA728599, MG-RAST identifier 4517724.3

^4^We run the species, genus, and family level datasets on a machine with a quad core Intel Core i7-6700K 4 GHz processor and 16GB of DDR4 RAM clocked at 2666 MHz. We run all other datasets on a machine with 1.5 TB of RAM and four Intel Xeon CPU E7-4830 v3 processors (48 total cores, 2.10 GHz each).
